# Treatment of Gastric Cancer

**DOI:** 10.3390/cancers12092627

**Published:** 2020-09-15

**Authors:** Stefano Rausei, Georgios D. Lianos

**Affiliations:** 1 Department of Surgery, ASST Valle Olona, Viale Eusebio Pastori 4, 21013 Gallarate, Italy; 2 Department of Surgery, University Hospital of Ioannina & University of Ioannina, 45500 Ioannina, Greece; georgiolianos@yahoo.gr

Surgery represents the only method for potentially curative intent for gastric cancer (GC). However, even after surgery, the prognosis of locally advanced GC remains dramatically poor. Five-year survival rates with resection alone range from 23% to 49% among Western and approximately to 70% in the Eastern countries [1,2]. These variations have been attributed to several differences between East and West regarding cancer biology, earlier diagnosis through screening programs, and surgeons’ compliance for a more extended surgery [3]. 

During the last two decades, it has been gradually accepted by the scientific community that GC patients should be treated by a multimodal and individualized approach [4]. However, the definition of the ‘optimal’ approach for GC patients is still debated and under extensive discussion.

Firstly, the treatment of GC has undoubtedly evolved with the recognition of the prognostic value of extended surgery, in terms of lymphadenectomy and multivisceral resection as well. On the other side, patient-friendly minimally invasive approaches along with enhanced recovery postoperative protocols have attracted scientific attention in GC management.

What is more, in the current century, randomized clinical trials demonstrated that chemotherapy or chemoradiation administered before, after, or both before and after surgery improve prognosis of GC patients [4].

After the long-debated discussion in the last century about the efficacy of extended lymphadenectomy, the results of a Taiwanese trial, published in 2006, have seemed rigorous enough to be considered definitive [5], along with the amount of consistent (though less strong) previous studies.

Now, the latest improvements in surgical techniques and increased surgeon experience make a patient-friendly minimally invasive surgical approach an achievable target also in GC treatment [6,7] and the surgical feasibility and safety of laparoscopic D2 distal gastrectomy have been demonstrated [8,9]. However, surgical outcomes (and technical perplexities) of laparoscopic total gastrectomy remain highly controversial [7,10]. Therefore, waiting for definite evidence of minimally invasive surgery for the cure of GC, its advent, along with its postoperative benefits—further enhanced by fast-track protocols—stressed a shift towards more personalized treatment of GC, with the intention of achieving better treatment efficacy for each patient, for each tumor [7].

Actually, “personalized treatment” and “patients’ selection” are the key words for multimodal strategy to GC. In the 2000s, upfront surgery is no longer the gold standard for non-early cases. In the West, every locally advanced tumor (often assessed by staging laparoscopy) is an indication of neoadjuvant chemotherapy [4,11]; recently, even the far East introduced the neoadjuvant approach for every tumor with bulky nodes [12]. 

Two European prospective randomized trials (MAGIC, French FNCLCC/FFCD) comparing perioperative chemotherapy versus surgery alone have definitively introduced in the West this new important perspective of treatment for GC [13,14]. Additionally, the recent results from the FLOT4 trial [15] report that a FLOT regimen can be used as a safe and efficient new standard regimen in the perioperative treatment of resectable GC. Consistently with these data and with the efficacy demonstrated for S-1-based schedules in the adjuvant setting, almost surprisingly, in the East, the SPIRITS study showed the possibility to candidate to curative surgery patients affected by GC with extensive nodal involvement after preoperative chemotherapy [16].

It is out of doubt that there is a lot of experience nowadays with neoadjuvant therapy and the neoadjuvant concept has become widely accepted; most research is now focused on the identification of the best regimen. However, we feel cautious acceptance might be more prudent [17].

On other hand, it is not to be excluded that even some metastatic GCs could sensationally respond to a first-line chemotherapy up to a successful, curative conversion surgery [18].

Actually, the future success of any therapeutic strategy resides in anticipating the response to treatment and more generally, prognosis of GC patients. Molecular classification of GC could provide potential novel pathways for patient stratification and trials of targeted therapies aiming to improve survival from this deadly disease [19].

While possibly, the ‘match’ against GC is definitely playing in molecular laboratories, all physicians are continuously investing time and resources to add quantity and quality to the life of every patient. This Special Issue aims to explore the areas of GC treatment remodeled or reconsidered with this perspective in the last two decades. Intriguing molecular insights, innovative drugs, new surgical conquests (and reconquests), technological advances, and modern clinical approaches are some of the topics we aim to focus on.
